# Discovery of four new B-cell protective epitopes for malaria using Q beta virus-like particle as platform

**DOI:** 10.1038/s41541-020-00242-y

**Published:** 2020-10-08

**Authors:** Erwan Atcheson, Gustavo Cabral-Miranda, Ahmed M. Salman, Arturo Reyes-Sandoval

**Affiliations:** The Jenner Institute, University of Oxford, Oxford, UK

**Keywords:** Vaccines, Peptide vaccines

## Abstract

Malaria remains one of the world’s most urgent global health problems, with almost half a million deaths and hundreds of millions of clinical cases each year. Existing interventions by themselves will not be enough to tackle infection in high-transmission areas. The best new intervention would be an effective vaccine; but the leading *P. falciparum* and *P. vivax* vaccine candidates, RTS,S and VMP001, show only modest to low field efficacy. New antigens and improved ways for screening antigens for protective efficacy will be required. This study exploits the potential of Virus-Like Particles (VLP) to enhance immune responses to antigens, the ease of coupling peptides to the Q beta (Qβ) VLP and the existing murine malaria challenge to screen B-cell epitopes for protective efficacy. We screened *P. vivax* TRAP (PvTRAP) immune sera against individual 20-mer PvTRAP peptides. The most immunogenic peptides associated with protection were loaded onto Qβ VLPs to assess protective efficacy in a malaria sporozoite challenge. A second approach focused on identifying conserved regions within known sporozoite invasion proteins and assessing them as part of the Qβ. Using this VLP as a peptide scaffold, four new protective B-cell epitopes were discovered: three from the disordered region of PvTRAP and one from Thrombospondin-related sporozoite protein (TRSP). Antigenic interference between these and other B-cell epitopes was also explored using the virus-like particle/peptide platform. This approach demonstrates the utility of VLPs to help identifying new B-cell epitopes for inclusion in next-generation malaria vaccines.

## Introduction

Malaria is one of the most urgent global health problems, with almost half a million deaths from *P. falciparum* and 7.5 million clinical cases of *P. vivax* malaria each year^1^. An effective vaccine could do more than any other intervention to help control and eliminate malaria. The leading *P. falciparum* vaccine, RTS,S, shows only ~30% efficacy under field conditions^2^ and VMP001, the most advanced *P. vivax* pre-erythrocytic vaccine, showed very low efficacy in a recent controlled human malaria infection trial^3^. New strategies and novel vaccine candidates will be essential to engineering an effective next-generation malaria vaccine.

The present study uses a highly immunogenic Virus-Like Particle (VLP), Qβ, to elicit strong antibody responses against peptides from malaria proteins, for screening as potential B-cell epitope targets of neutralising antibodies for further development as effective malaria vaccines. Peptide vaccines have a long history in malaria vaccine development^4–8^, but presentation on a virus-like particle offers the advantage of much-improved immunogenicity^9^, which increases the chances that even weak epitopes with protective potential can be identified.

Two approaches for identifying new B-cell epitopes with protective potential are pursued in this study. One is to screen sera from TRAP-vaccinated mice against peptides spanning the sequence to then select immunogenic peptides for coupling to Qβ for use in murine vaccination and challenge experiments. TRAP is a liver-stage antigen used by parasites to gain entry to hepatocytes prior to the blood stage of infection. The vaccine efficacy of TRAP, a liver-stage antigen, is known to be mediated by CD8^+^ T-cells^10–13^ but evidence also suggests a role for antibody-mediated protection^14–17^. A useful feature of the mouse model for malaria vaccine testing is the ability to use transgenic malaria parasites, based on the rodent *P. berghei* malaria, but expressing (in this case) TRAP from the human malaria parasite *P. vivax*, for more direct utility to clinical vaccine development.

The second method is based on the hypothesis that highly conserved regions of proteins are conserved because they play some functional role^18^. Thus, if conserved regions in proteins are synthesized as peptides, coupled to a virus-like particle such as Qβ and used to vaccinate animals to generate antibodies, and if those antibodies raised against the peptides are able to recognise that sequence in the context of the native protein, then those antibodies should have an increased probability of neutralising the sporozoite by virtue of their sterically interfering with the function of that protein. An increasing body of data is available on surface expression^19^ and the role in invasion of sporozoite proteins; a list used to generate candidate vaccines in this study is given below (Table 1).

**Table 1:** *P. berghei* proteins used for conservation-based screening of linear B-cell epitopes for protective efficacy.

Protein	Accession no.	Initial description	Evidence for role in liver-stage infectivity and as vaccine
PBLP Plasmodium BEM46-like protein	A0A113RBV9	^41^	^41^
SSP3 sporozoite surface protein 3	PBANKA_1425200	^42^	^42^
SIAP 1 sporozoite invasion-associated protein 1	PBANKA_1006200	^43^	^43,44^
Maebl merozoite adhesive erythrocytic binding protein	PBANKA_0901300.2	^45^	^46–49^
RON4 rhoptry neck protein 4	PBANKA_0932000	^50^	^51^
P113 surface protein P113	PBANKA_1022500	^52^	^53^
PLP1 sporozoite micronemal protein essential for cell traversal	PBANKA_1006300	^54^	^55–57^
RON3 rhoptry neck protein 3	PBANKA_1464900	^58^	none^a^
Falstatin	PBANKA_0813000	^59^	^60–62^
TRSP Thrombospondin-related sporozoite protein	A1X5V6-1	^63^	^64^

^a^Evidence only for RON3 surface expression on sporozoites^19^.

The goal of both approaches is to discover new B-cell epitopes with potential to be further developed as malaria vaccines, exploiting the ease of use of the Qβ-peptide platform with malaria challenge experiments in mice as a medium-throughput malaria vaccine screening system.

## Results

### Identification and protective efficacy of linear B-cell epitopes in *P. vivax* TRAP

**Fig. 1:**
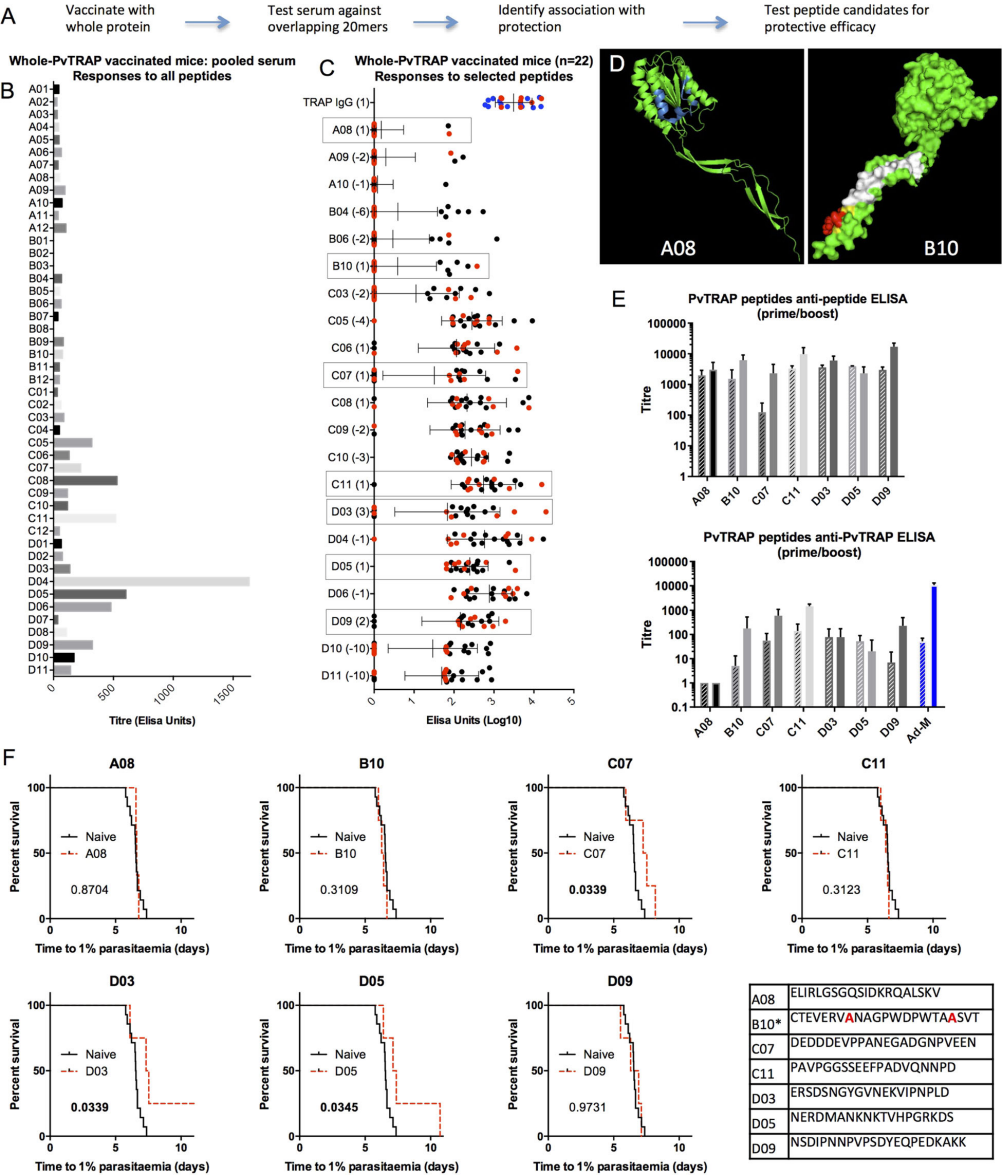
Identification of neutralising linear B-cell epitopes in *P. vivax* TRAP. **a** Schematic of screening strategy. **b** Pooled sera from BALB/c mice vaccinated with PvTRAP (viral vectors, adenovirus prime and MVA boost, eight week prime-boost interval) was used to screen responses to overlapping 20-amino acid peptides spanning the length of the protein using standard curve ELISAs. **c** Sera from 21 mice vaccinated as in (B) were used to determine responses to 21 selected PvTRAP peptides by standard curve ELISAs. Also shown (“TRAP IgG”) are serum responses to whole PvTRAP. Mice that showed a delay in time to reach 1% blood-stage parasitaemia are coloured red, and those showing no evidence of vaccine effect are coloured black. Numbers in brackets after the peptide label indicate the number of protected mice showing a titre greater than that of the highest-titre unprotected mouse; or, for negative numbers, the number of unprotected mice showing a greater titre than that of the highest-titre protected mouse. **d** Locations (in red) of the indicated peptides on partial PvTRAP crystal structure. B10 is represented in white apart from the conserved CSVTCG domain, in red, and cysteines yellow (4HQ0). **e** Selected peptides from **c** were individually coupled to Qβ and used to vaccinate BALB/c mice (1 µg per dose, three shots at three-week intervals, with Matrix-M™ adjuvant). Standard curve ELISAs against homologous peptides and against whole PvTRAP were performed. Also shown (Ad-M) are responses to adenovirus-primed and MVA-boosted viral-vectored PvTRAP, in blue. Post-prime titres shown in hatched bars, post-boost solid. **f** Mice from **e** were challenged three weeks post-final vaccination by intravenous injection of 1000 PvCSP/PvTRAP transgenic sporozoites, and time to 1% parasitaemia determined by linear regression from thin blood smears taken daily. Numbers indicate p-values from log-rank tests compared to naïve. Also shown are the peptide sequences displayed on Qβ vaccines. For B10, Asterisk indicates that cysteines were exchanged for alanines, marked bold red.

Linear B-cell epitopes in *P. vivax* TRAP (PvTRAP) were identified by screening peptide ELISA responses to PvTRAP 20mer peptides spanning the entire length of PvTRAP (Fig. 1a, b), using sera from viral-vectored PvTRAP-vaccinated mice. 21 immunogenic peptides were selected for further ELISAs using sera from 21 PvTRAP viral-vector vaccinated mice, on the basis of their immunogenicity. Seven of these were further selected for chemical coupling to Qβ on the basis of possible, though not statistically significant, associations with protection (Fig. 1c); for example, D08 despite being immunogenic was at its highest titre non-protective. Two of these peptides are located on the crystal structure of PvTRAP (Fig. 1d). All Qβ-peptide vaccines generated high levels of antibody against the corresponding peptide, and all but one (Qβ-A08) generated antibodies recognising native PvTRAP at levels comparable to those elicited by viral-vectored PvTRAP vaccination (Fig. 1e). Three of the seven Qβ-peptide vaccines conferred statistically significant partial protection against challenge with 1000 transgenic *PvCSP-210/PvTRAP P. berghei* sporozoites: C07 (*p* = 0.0339), D03 (*p* = 0.0339) and D05 (*p* = 0.0345) (Fig. 1f).

**Fig. 2:**
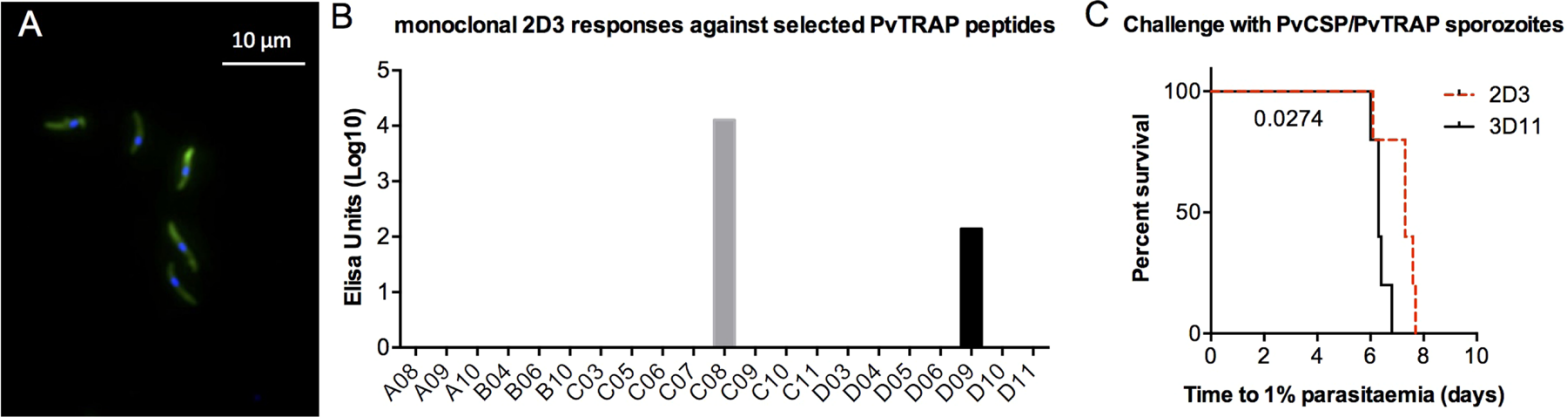
2D3 PvTRAP monoclonal antibody specificity and neutralising potency. The 2D3 monoclonal antibody was obtained by limiting dilutions of hybridomas derived from PvTRAP-vaccinated mice. **a** 2D3 recognises PvTRAP-expressing *P. berghei* in an immunofluorescence assay (green: 2D3; blue: Hoechst DNA stain). **b** ELISAs using 2D3 were performed against selected peptides from the PvTRAP sequence. **c** PvCSP/PvTRAP transgenic *P. berghei* sporozoites were incubated at room temperature for 30 min with 190 µg/mL of either PbCSP-specific 3D11 mAb or 2D3, then BALB/c mice (*n* = 5 per group) were challenged by intravenous injection of 1000 sporozoites each. Time to reach 1% blood-stage parasitaemia was calculated by linear regression from thin blood smears taken daily. Number represents *p*-value from log-rank test.

All three peptides lie in the disordered region of PvTRAP. Further evidence that the disordered region of PvTRAP is a target of neutralising antibodies comes from an experiment with a monoclonal PvTRAP antibody, 2D3, which recognised *PvCSP/PvTRAP* sporozoites (Fig. 2a) and showed responsivity to peptides in the disordered region of PvTRAP (Fig. 2b). PvCSP/PvTRAP sporozoites were incubated with either 2D3 or the PbCSP-specific 3D11 control mAb prior to injection into mice and 2D3 conferred partial protection compared to control-treated mice (*p* = 0.027) (Fig. 2c).

### Identification and protective efficacy of conserved linear B-cell epitopes within invasion proteins

So far *P. vivax* transgenic sporozoites were used to test *P. vivax-*based vaccines, but for the remainder of the experiments here reported *P. falciparum*-based sporozoites were used for challenge studies, because the epitopes here examined were conserved between species and because interest in combining these with existing *P. falciparum* vaccines (RTS,S or the NANP epitope in PfCSP) is likely to be high. Pre-clinical tests of such combinations will be the focus of further studies.

**Fig. 3:**
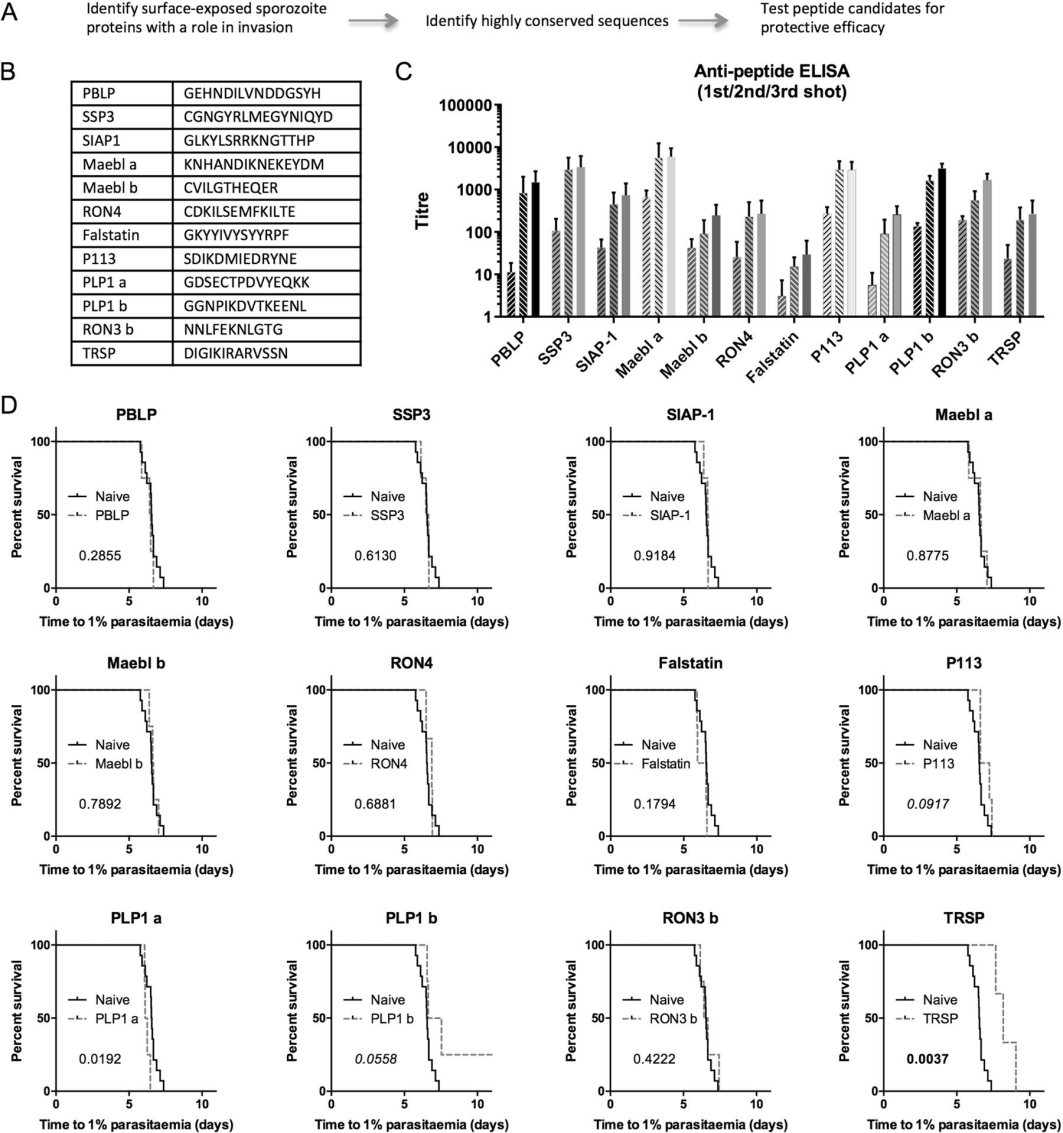
Identification of neutralising linear B-cell epitopes by screening conserved peptides in *P. berghei*. The strategy shown in **a** was used to identify conserved amino acid sequences in the sporozoite surface proteins shown in **b**. The peptides were chemically coupled to Qβ and used to vaccinate BALB/c mice (three doses, 1 µg per dose, at three-week intervals, with Matrix-M™ adjuvant). **c** Sera was taken 2 weeks post-vaccination and used in standard curve ELISAs against the homologous protein. Titres after first shot shown upwards-right hatch; after second shot downwards-right hatch; after third shot solid. **d** Mice were challenged three weeks after the final vaccination by intravenous injection of 1000 PvCSP/PvTRAP transgenic *P. berghei* sporozoites. Time to 1% parasitaemia was calculated by linear regression from daily thin blood smears. Numbers represent *p*-values from log-rank tests in comparison to naïve.

On the hypothesis that antibodies binding conserved sites within invasion proteins may be neutralising by virtue of interfering with protein function, 12 candidate peptides based on conserved regions from ten malaria sporozoite invasion proteins were selected as Qβ-peptide candidates (Fig. 3a, b). To this end, we identified proteins using PlasmoDB, aligned them and identified conserved regions of these proteins, to finally synthesize peptides of 16–20 amino acids and load them onto Qβ. The Qβ-peptide vaccines were used to vaccinate BALB/c mice. All peptides except the Falstatin peptide were immunogenic against the corresponding peptide (Fig. 3c). Challenge of vaccinated mice with 1000 *P. berghei* sporozoites showed one candidate Qβ-peptide conferring a significant degree of protection on mice, using a peptide derived from TRSP (*p* = 0.0037) (Fig. 3d). Two other candidates P113 (*p* = 0.09) and PLP1b (*p* = 0.056, with one case of sterile protection) showed borderline efficacy (Fig. 3d).

**Fig. 4:**
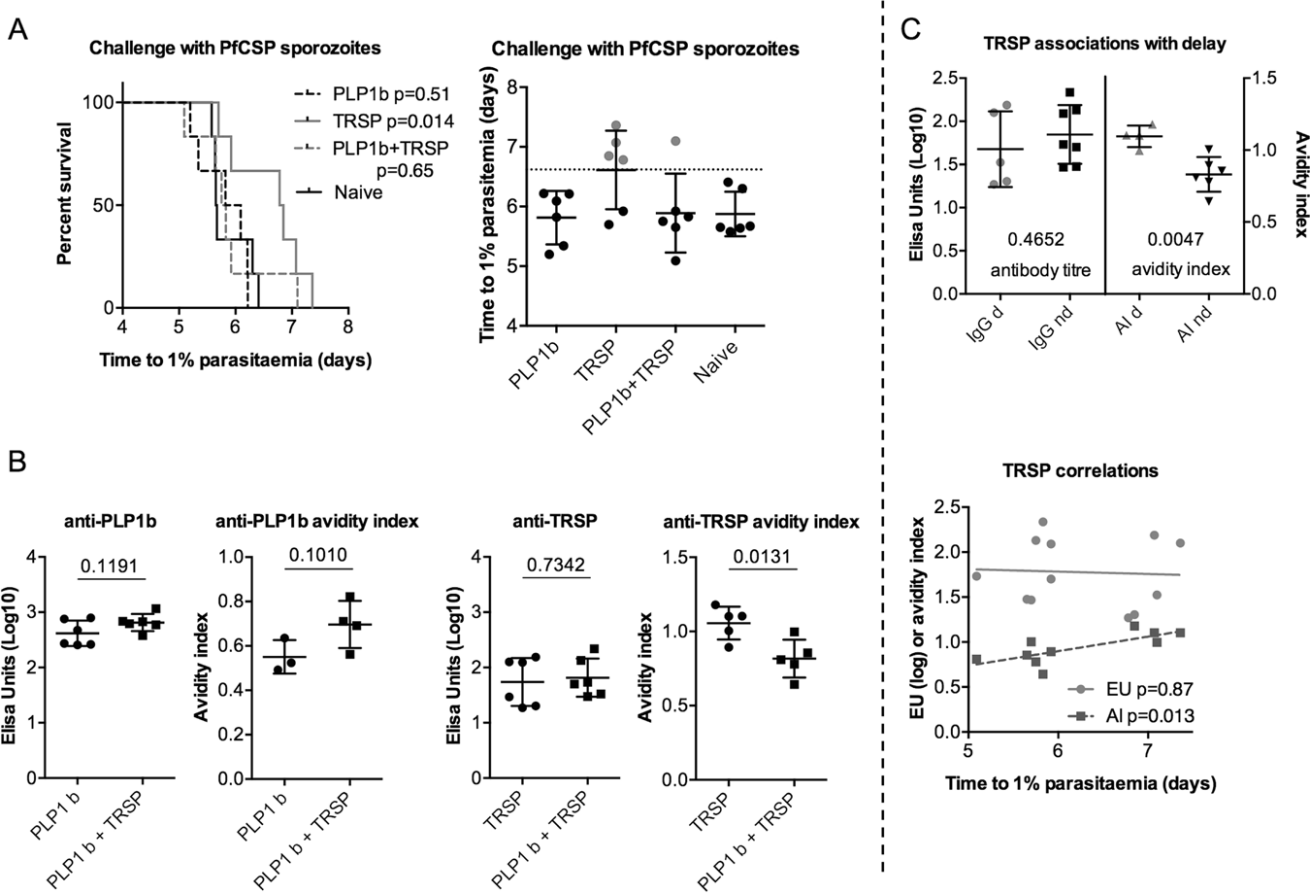
Effects on immunogenicity and protective efficacy of combining Qβ-(PLP1b) and Qβ-(TRSP) vaccines. BALB/c mice (*n* = 6 per group) were vaccinated with two shots by intramuscular injection of 1 µg of each Qβ-peptide in Matrix-M™ adjuvant using a three-week prime-boost interval. Qβ-(PLP1b) was delivered into the left leg and Qβ-(TRSP) into the right leg. **a** Mice were challenged three weeks post-boost by intravenous injection of 1000 PfCSP replacement *P. berghei* sporozoites into the tail vein. Time to reach 1% blood-stage parasitaemia was calculated by linear regression from thin blood smears taken daily. Numbers represent p-values from log-rank tests between indicated groups. The column figure indicates which mice show a delay in time-to-1%, coloured grey, defined as time-to-1% greater than the mean naïve time-to-1% plus two standard deviations; this is indicated by the hatched line. **b** Standard curve ELISAs from sera taken two weeks post-boost, with avidity index calculated as the ratio of titres from sera treated to untreated with 7 M urea. Numbers represent p-values from ANOVA with Bonferroni’s multiple comparisons test. **c** Mice were divided into two groups, delayed (“d”) or no delay (“nd”) as shown in **a**; *p*-values shown below groups indicate strengths of association (by *t*-test) with either titre or avidity index (AI). Correlations between time-to-1% and either titre (EU) or avidity index (AI) also shown; *p*-values derived from *F*-test.

An experiment was carried out to determine if the enhancement of protective efficacy could be obtained by combining Qβ-(PLP1b) and Qβ-(TRSP). Qβ-(TRSP) again demonstrated protective efficacy, but Qβ-(PLP1b) did not (Fig. 4a). Interestingly, the combination showed a lower protective efficacy than Qβ-(TRSP) alone. The effect of Qβ-(PLP1b) on anti-TRSP titres was minimal, but did significantly reduce the affinity of anti-TRSP antibodies (Fig. 4b), with affinity and not titre of anti-TRSP showing a significant association with protection (Fig. 4c).

### Investigation of immune hierarchies of linear malaria B-cell epitopes

**Fig. 5:**
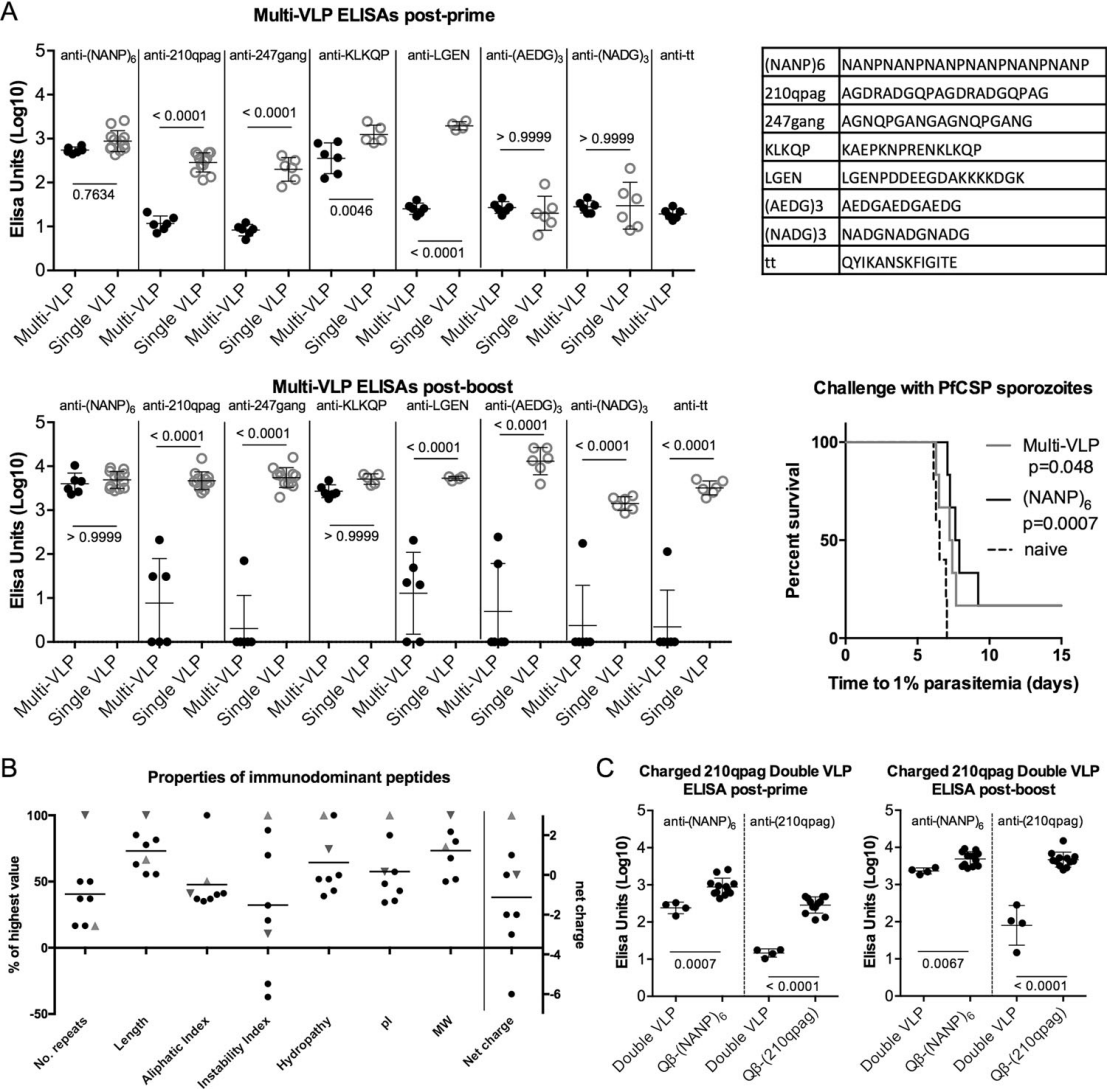
Immunodominance on a multi-epitope VLP. A Qβ-peptide vaccine with eight epitopes chemically coupled simultaneously (“Multi-VLP”) was produced using the peptides shown in **a**. BALB/c mice were vaccinated by intramuscular injection (3 µg per dose, 3-week prime-boost interval, with Matrix-M™ adjuvant) using either the Multi-VLP or one of eight single-epitope Qβ VLPs displaying one of the peptides. **a** Standard curve ELISAs were performed on sera taken 2 weeks post-vaccination against the indicated peptides. Numbers on ELISA graphs show *p*-values from ANOVA with Bonferroni’s multiple comparisons test. Mice were challenged three weeks after the final dose with 1000 PfCSP transgenic *P. berghei* sporozoites and thin blood smears taken daily to determine time to reach 1% blood-stage parasitaemia by linear regression. *P*-values are from log-rank tests compared to naive. **b** The properties of the two immunodominant epitopes, (NANP)_6_ (downwards triangle) and KLKQP (upwards triangle), were compared to the others. Values of each property are expressed as percent of the highest value in each case, except for net charge. pI: isoelectric point. MW molecular weight. **c** Three lysine residues were added to 210qpag to increase the net charge and this was chemically coupled simultaneously with (NANP)_6_ to Qβ to create a “Double VLP”. Mice were vaccinated and ELISAs performed as before. Numbers represent *p*-values from *t*-tests.

Immune interference between peptides could represent a major obstacle to successfully combining malaria vaccines. To shed further light on the phenomenon, a single-component multi-epitope vaccine (“Multi-VLP”) was produced by simultaneously coupling eight epitopes to Qβ (Fig. 5a). BALB/c mice were vaccinated using this Multi-VLP and immune responses compared to mice vaccinated with single-epitope Qβ-peptides. (NANP)_6_ presented on the Multi-VLP was immunodominant, with titres unaffected either post-prime or post-boost in comparison to Qβ-(NANP)_6_ (Fig. 5a). This was reflected in a challenge experiment where both the Multi-VLP and Qβ-(NANP)_6_ conferred similar levels of protective efficacy (Fig. 5a). Post-boost, anti-(KLKQP) titres were also undiminished by Multi-VLP vaccination in comparison to Qβ-(KLKQP) vaccination, making (NANP)_6_ and (KLKQP) co-dominant epitopes. In contrast, antibody responses to the other six epitopes were greatly diminished, by about three orders of magnitude, (*p* < 0.001) when the peptides were presented on the Multi-VLP compared to single-peptide Qβ VLPs (Fig. 5a). The pattern of immunodominance was not identical post-prime and post-boost: post-prime, anti-(KLKQP) titres were slightly but significantly lower using Multi-VLP, whereas anti-(AEDG)_3_ and anti-(NADG)_3_ were not abrogated post-prime. This demonstrates that the immune hierarchy post-prime does not necessarily predict the hierarchy of immunodominance we will see post-boost.

A survey of the properties of the peptides coupled to Qβ, including the number of repeats, number of amino acids (length), aliphatic index, instability index, hydropathy, pI, molecular weight and net charge, was carried out to identify potential explanations for the immunodominance of (NANP)_6_ and (KLKQP) (Fig. 5b). (NANP)_6_ has the greatest number of repeats, molecular weight and length. (KLKQP), in contrast, is short and not repetitive. However, (KLKQP) has the highest instability index, hydropathy, pI and net charge. It has yet to be determined which if any of these factors lead to (KLKQP) being co-dominant with (NANP)_6_ at the expense of the other epitopes. An experiment was carried out to see if increasing the net charge of the 210qpag peptide by addition of three lysine residues would prevent immune interference when this positively charged 210qpag peptide was simultaneously coupled with (NANP)_6_ to Qβ (“Double VLP”). Anti-210qpag titres remain abrogated using the Double VLP post-prime and post-boost (*p* < 0.0001, one to two orders of magnitude difference compared to Qβ-210qpag). In this case anti-(NANP)_6_ titres were also significantly lower when vaccinating with the Double VLP compared to Qβ-(NANP)_6_ (post-prime, *p* = 0.0007, post-boost *p* = 0.0067) (Fig. 5c).

## Discussion

Four new B-cell epitopes have here been discovered, each capable of conferring partial protection against malaria as peptides presented on a virus-like particle. The epitopes were discovered using two techniques: screening sera for peptides recognised by antibodies elicited by whole protein vaccination or exploiting conservation within sporozoite invasion proteins. The utility of the Qβ-peptide platform is thus demonstrated, establishing medium-throughput screening of peptides as potential protective epitopes for further development, pre-clinically and clinically, as malaria vaccine candidates. Though none of the peptide vaccines in this study shows high levels of protection, the utility of these approaches will be in quickly and reliably discovering new protective B-cell epitopes susceptible to further improvement as parts of highly protective vaccines. The sensitivity of the platform to even low levels of protective efficacy is well-established^11^, and is an advantage of the approach, although variations in (for instance) parasite infectivity between challenge experiments means direct comparison between different experiments is often not possible^20^. The Qβ-peptide platform is also of use for pre-clinical screening of epitopes that will be co-dominant, avoiding antigenic interference, a problem likely to be of increasing importance as single subunit vaccines are replaced by more complex combination strategies^20–22^.

Screening sera of animals immunized with a putative malaria vaccine candidate (PfSEA) against short peptides has previously been used to generate information about linear B-cell epitopes associated with protection^23^, but in the present study, that technique was extended to down-select linear B-cell epitopes as vaccine candidates tested for protective efficacy in a challenge experiment, using whole PvTRAP as the original immunogen. Of seven PvTRAP peptides selected, three showed clear evidence of partial protective efficacy. All three peptides derive from the apparently disordered region C-terminal of the von Willebrand factor A and TSR domains^24,25^, as apparently do the epitopes recognised by the neutralising monoclonal antibody 2D3. It will be of interest in further experiments to determine if the C08 peptide recognised by 2D3 also confers protection presented on Qβ. Interestingly, these and other disordered regions in malaria proteins are predicted to have an abundance of linear B-cell epitopes^25^, which was verified here for TRAP. The same study classifies the repeat region of circumsporozoite protein as highly disordered, significant as this too is a target of neutralising antibodies and forms the core of the leading malaria vaccine RTS,S. Further study should investigate whether disordered regions of malarial proteins, in general, may be good candidates for identifying neutralising linear B-cell epitopes by screening Qβ-peptide vaccines in pre-clinical challenge experiments.

The exact mechanism of action of the PvTRAP-peptide vaccines here identified is not known, but the identified peptides do either encompass or lie next to conserved motifs in the PvTRAP sequence. C07 lies just C-terminal of an LDVPDE motif common to *P. falciparum*, *P. vivax* and several other species of malaria. D03 and D05, contiguous in the PvTRAP protein sequence, between them, straddle an LDNER motif shared by *P. vivax, P. falciparum* and *P. reichenowi*. As conserved regions of proteins often play a functional role^18^, it is plausible that the antibodies elicited by these peptides are neutralising by virtue of interfering with some function of PvTRAP in this region. It will be interesting to see if the PvTRAP peptides are able to elicit cross-species immunity, or if the protection is species-specific. A multiple antigenic peptide study has used short peptide sequences (DRYI and TRPHGR) just C-terminal of the equivalent region to D05 in *P. falciparum* TRAP as vaccines^26^. This study found that antisera from mice so vaccinated and monoclonal antibodies recognising these peptides could inhibit sporozoite invasion in vitro. This underscores the potential of this extended region as a vaccine candidate and should be the focus of further study; although the efficacy conferred by individual peptides is low, extending vaccination to include all (and only) the disordered region of TRAP may be more protective, focusing the response on neutralising targets and away from immunogenic but antigenically non-protective regions of TRAP. It will also be of interest to try combining the immunodominant CD8^+^ T-cell epitope from PvTRAP with protective PvCSP repeat peptides^27^ on a single immunodominant antigen, although research has shown that much higher doses of Qβ are required^28^.

The second screening strategy used in this chapter exploited conservation directly. Conserved regions of proteins are often associated with function^18^. Thus, antibodies elicited against these regions may be neutralising by virtue of interfering with that function. To our knowledge, this technique has never before been exploited as a means of generating protective vaccines. Of the twelve peptides, generated from the conserved regions of *P. berghei* sporozoite invasion proteins, most were highly immunogenic as Qβ-peptide vaccines, but only one, derived from a conserved TRSP sequence, demonstrated clear evidence of protective efficacy. The TRSP epitope here identified as a target of neutralising antibodies lies immediately downstream of the highly conserved EWSQCSKTC motif present (with variations) in the TSR domains present in CSP and TRAP and implicated in binding to hepatocytes and invasion^29–32^. What is most interesting is that this epitope represents a short Region II epitope that actually appears to elicit neutralising antibodies, unlike those from CSP^33–35^. A previous study has found that the positively charged residues downstream of the CSVTCG motif in CSP mediate binding to hepatocytes, probably via negatively charged glycosaminoglycan residues^36^. The TRSP peptide contains these basic residues (bold): DIGI**K**I**R**A**R**VSSN. This suggests that peptides based on the positively charged glycosaminoglycan-binding residues C-terminal of the CSVTCG motifs in CSP and TRAP might also act as good vaccine candidates, especially if they omit the CSVTCG motif altogether, as the cysteines may interfere with the conformation of the peptide, as is the case with many cysteine-containing proteins.

A reduction in the affinity of anti-TRSP epitope antibodies when Qβ-(TRSP) was combined with Qβ-(PLP1b) ablated the protective efficacy of that vaccine. This demonstrates that the principal challenge facing attempts to make a multi-antigen vaccine will be antigenic interference. Interestingly, the co-dominance seen between a KLKQP-epitope and (NANP)_6_ is not compatible with the only current model of B-cell epitope immunodominance^37^, and gives reason to hope that multi-antigen vaccines can be designed to avoid immunodominance by any one B-cell epitope. An attempt to use extra positive charge to overcome immunodominance was explored in our study, but did not demonstrate any improvement. Altering the length or relative density of epitopes may see greater success and could be the first line of attack in dealing with this problem in future studies. It would also be of interest to see if the findings from this study, performed in BALB/c mice, also hold when other strains are used.

The utility of the Qβ-peptide platform for carrying out such exploratory studies, as well as for investigations of the vaccine potential of B-cell epitopes, is clear.

## Methods

### Vaccination

Isofluorane-anaesthetised mice were vaccinated by intramuscular injection (25G needle) of 25 μL vaccine formulation into left and right hind muscles, with 3-week prime-boost intervals between doses. Matrix-M™ adjuvant (Novavax AB, Uppsala, Sweden) was used at 12 µg per dose. Adjuvants were kindly provided by Dr. Anita Milicic from the Jenner Institute adjuvant bank. Viral-vectored PvTRAP was administered as a heterologous prime boost, with simian adenoviral vector 63 (ChAd63) given at 10^8^ infectious units followed by modified vaccinia virus strain Ankara (MVA) at 10^7^ plaque-forming units per dose 8 weeks later. Qβ-peptide vaccines were given at a dose of 1 µg per dose.

### Mouse strains used

6 week-old female BALB/c (H-2^d^) mice were used for vaccination experiments, with age-matched controls. TO outbred mice and BALB/c mice were used for parasite maintenance and mosquito feeds. All mice from Harlan/Envigo.

### Ethics statement

All animals and procedures were used in accordance with the terms of the United Kingdom Home Office Animals Act Project License. The procedures were approved by the University of Oxford Animal Care and Ethical Review Committee (PPL 30/2889 and P9804B4F1).

### Infection of *Anopheles stephensi* mosquitoes with *P. berghei*

Cryopreserved mouse blood stocks of wild type or transgenic *P. berghei* from liquid nitrogen were defrosted and immediately administered to naïve BALB/c or TO mice by intraperitoneal injection (100 µL). Thin blood smears were taken daily and when gametocytes were observed mice were anaesthetised by intramuscular injection (Rompun/Ketaset) for mosquito feed. Mosquitoes starved for 2 h were allowed to feed for 10–15 m on anaesthetised infected mice. Blood was taken from mice to confirm exflagellation of gametocytes by microscopy. After feeding, mosquitoes were returned to fructose/P-amino benzoic acid on cotton wool and maintained in the Jenner Institute insectary (19–21°C, 12 h light/dark cycle). One week after feeding a second feed was performed on an anaesthetised naive mouse to improve sporozoite yields. Mosquitoes were maintained for a total of 21 days prior to dissection of sporozoite-infected salivary glands.

### Dissection of mosquito salivary glands and challenge of mice with sporozoites

Twenty-one days after feeding on *P. berghei* infected mice, mosquitoes were sedated at 4 °C for dissection. Salivary glands were dissected from mosquitoes under a microscope and removed by pipette into a glass tissue homogeniser containing 100 µL Schneider’s insect media with 10% FBS. Sporozoites were liberated from salivary glands by gently homogenising three times and counted using a haemocytometer. Sporozoite concentration was adjusted to 10^4^ sporozoites/mL for intravenous injection into the tail vein of mice of 100 µL (1000 sporozoites per dose, by insulin syringe).

### Thin blood smears and calculation of time to reach 1% blood-stage parasitaemia

Daily thin blood smears were prepared on glass slides from a drop of blood taken from the tail tip of challenged mice. Slides were fixed in methanol then stained in 5% Giemsa (Sigma) for 30 min and washed in water. In all, 1000 red blood cells were counted for three to five consecutive days starting on day 4. For extrapolation of the liver-to-blood parasite load and to predict the time taken to reach 1% blood-stage infection, a linear regression model was used and Log_10_ of the calculated percentage of parasitaemia was plotted against time after challenge, using Prism 6 for Mac OS X (GraphPad Software) for generating a linear regression model on the linear part of the blood-stage growth curve^11^. Mice without parasites by day 15 were considered to have been conferred sterile protection against challenge.

### Transgenic *P. berghei* parasites

We used for challenge, *P. berghei* parasites expressing PvCSP VK210 and PvTRAP in place of endogenous PbCSP and PbTRAP^22^, or PfCSP 3D7 in place of PbCSP^38^. Accession numbers for each *P. berghei* sequence are provided (Table 2).

**Table 2:** Replacement transgenic *P. berghei* and accession numbers.

Parasite name	Transgene	Accession number
Pv-CSP-210/PvTRAP	*PvCSP-210*	PVX_119355
	*PvTRAP*	XP_001614147.1
PfCSP	*PfCSP [3D7]*	Q7K740

### Expression of proteins in HEK293 cells and purification

PvTRAP protein was expressed by cloning the gene of *P. vivax* TRAP with additional codon optimization for mammalian cell expression (UniProt A5K806, residues Asp25-Lys493) into the pHLsec vector. The protein was expressed by transient transfection in HEK-293T cells and purified after dialysis with PBS buffer, using conditioned medium by immobilized Co^2+^-affinity chromatography and size-exclusion chromatography in 20 mM Tris-HCl pH 8.0, 300 mM NaCl^39^.

### Qβ virus-like particle production, purification and chemical coupling

Qβ-transformed *E. coli* from glycerol stock was grown to 1 mL in LB/carbenicillin, then transferred to 1 L M9 media (with 2 mL MgSO_4_, 5 mL 40% glucose, 50 mL casamino acid, 500 µL vitamin B1, and 100 mg/mL carbenicillin) and incubated at 37°C 250 rpm for 18 h. Cells were pelleted (4500 rpm, 25 min, 4 °C) and the supernatant discarded. The pellet was resuspended in PBS, centrifuged again (20 min, 14,000 g), and supernatant discarded. The pellet was lysed using lysis buffer (20 mM NaPO_4_ pH 7.5, 0.1% triton x-100, 5 mM EDTA, 100 U/g cells Benzonase, 10 µL/g cells Lysonase, 10 µL/ml protease inhibitor), and freeze/thawing the pellet in dry ice twice. Lysed cells were sonicated for 1 min (15 s on/30 s off, 30% intensity), centrifuged at 14,000 g for 25 min, and the supernatant collected and filtered. Fractogel purification was carried out using 20 mM NaPO_4_ pH 7.2 buffer with either 150 mM or 1 M NaCl, followed by size exclusion chromatography.

Coupling Qβ peptides were performed by derivatising Qβ with reactive groups using succinimidyl-6-[(β-maleimidopropionamido)hexanoate] (SMPH) at 10X molar excess SMPH (1 h, 250 rpm RT), followed by three 1 m 100 kDa spin filtrations with PBS (Amicon 0.5 mL) to remove free SMPH. Peptides were synthesised with free cysteines rendering SATA derivation unnecessary. Peptides were incubated with SMPH-derivatised Qβ for 3 h (250 rpm, RT) and Qβ-VLPs stored at −20 °C. All peptides were synthesised by ThinkPeptides.

### ELISAs: standard curve, affinity

Nunc Maxisorp 96-well plates (Sigma) were coated with antigen (50 µl, 1 µg/mL in PBS) and incubated overnight at RT. Plates were washed six times with PBS/0.05% Tween (PBS/T) (Sigma) and blocked for 1 h with 10% skimmed milk (Sigma) in PBS/T (100 µL/well). Microvette serum tubes (Sarstedt) were used to collect blood from tail veins of mice and serum obtained by centrifugation (13,000 rpm, 10 min). Sera was typically diluted at 1:500 post-prime, 1:1000 post-second shot and 1:2000 post-third shot and applied to plates in triplicate after blocking (2 h RT incubation). Standard curves were prepared on each plate against the antigen of interest by serial dilution of standard sera obtained by cardiac bleed from mice vaccinated with the specific antigen being tested in ELISA. Plates were washed as before and goat anti-mouse whole IgG alkaline phosphatase conjugate (Sigma) applied (50 µl/well, 1:5000 in PBS/T, 1 h RT). Plates were washed as before and 1 mg/mL pNPP (Sigma) in diethanolamine buffer (Pierce) applied to the plates (100 µl/well) and allowed to develop with readings on a BioTech Microplate Reader taken at 14 min and 1 h at 405 nm. Titres were expressed as arbitrary ELISA units (EU) relative to a standard curve.

To determine the avidity index, a replicate ELISA was performed identical to and simultaneously with the standard curve ELISA, except that after 2 h incubation with diluted sera, 100 µL 7 M urea (Sigma) was applied to each well for 10 min (excluding the standard curve). Plates were then washed and the ELISA completed as before. The avidity index is the ratio of urea-treated to untreated ELISA units^40^.

### Hybridoma and monoclonal 2D3 PvTRAP antibody production

Sp2/0ag14 cells were grown in DMEM with 10% FBS for two weeks, with 8-azaguanine (Sigma) added during the first week. One day prior to fusion macrophages were obtained from a naïve mouse and cultured in DMEM with HAT, HT and 10% FBS and penicillin/streptomycin. The following day the spleen from a mouse vaccinated with recombinant PvTRAP was removed, crushed in 5 mL PBS, passed through a 70 µm cell strainer, 10 mL PBS added, followed by centrifugation (1350 rpm for 5 min), resuspension of the cell pellet, treatment with 5 mL ACK for 5 min, then addition of 25 mL PBS and centrifugation as before. The supernatant was discarded and cells resuspended in DMEM and counted using a haemocytometer. Sp2/0ag14 cells were gently mixed 1:10 with spleen cells (~2 × 10^7^:2 × 10^8^ cells) then centrifuged 10 min at 500 g RT. The supernatant was discarded and pellet loosened by flicking. In all, 800 µl 50% PEG (MW 1500) at 37 °C with 7.5% DMSO was added drop-by-drop over one minute in a water bath. 30 mL 37°C DMEM was then added drop by drop over 15 min followed by 10 min incubation. Cells were centrifuged for 5 min 500×*g* RT and the supernatant discarded. In all, 48 mL DMEM/10% FBS/HAT/HT/pen./strep. was added and 500 µL/well applied to 24-well plates containing macrophages and incubated for 5 days (37 °C, 5% CO_2_), whereupon 500 µL of media was removed and replaced, with this process continuing until cells were growing and supernatant could be tested for the presence of PvTRAP-specific antibody by ELISA.

Wells testing positive for PvTRAP antibody by ELISA were selected for cloning by limiting dilution. Cells were resuspended in cloning medium DMEM/4 mM L-glutamine/20% FBS/10% Hybridoma Cloning Factor, counted and viability determined to be over 80%. Serial dilution of cells to 4, 2 and 1 cells/mL in cloning medium was performed, plated into 96-well plates and incubated at 37 °C 8–10% CO_2_ for 5–7 days. Wells containing cells were tested for monoclonal antibody by ELISA as before. One monoclonal antibody was obtained: 2D3.

2D3 and 3D11 monoclonals (3D11 is a PbCSP repeat region mAb, hybridoma obtained courtesy of Julie Furze) were obtained from hybridomas by propagation in DMEM/pen/strep/L-glutamine/20% ultra-low IgG FBS and incubated at 37 °C 5% CO_2_. The supernatant was removed and mAb purified using a Pierce Protein G column, with mAb buffer exchanged into PBS using a 30 kDa MWCO Millipore centrifugal filter unit.

### PvTRAP crystal structure visualisation

The PvTRAP crystal structure (4HQO)^24^ was visualised and coloured using PyMol.

### Calculation or prediction of peptide properties

Length, net charge, aliphatic index, instability index and hydrophobicity were calculated or predicted using the CAMP.R3 feature calculator (http://www.camp3.bicnirrh.res.in/featcalc). Molecular weight and theoretical pI were calculated using the ExPasy ProtParam tool (web.expasy.org/protparam).

### Selection and identification of conserved amino acid sequences in *P. berghei* pre-erythrocytic invasion proteins

A list of surface-exposed sporozoite proteins involved in invasion and migration^19^ was used as the starting point for identifying conserved regions of *Plasmodium* invasion proteins for the design of peptides to act as vaccine candidates. Homologues of selected proteins were identified by protein BLAST using PlasmoDB (plasmodb.org), Uniprot and NCBI. Alignments to identify conserved regions were performed using Clustal Omega (www.ebi.ac.uk/Tools/msa/clustalo/) and transmembrane domains predicted using TMHMM Server 2.0 (www.cbs.dtu.dk/services/TMHMM/) to identify likely extracellular regions of proteins. Conserved regions of these proteins were identified visually and used as the basis for choosing 16–20 amino acid sequences for synthesis. Table 3 gives the accession numbers for each protein used in each alignment.

**Table 3:** Accession numbers of *Plasmodium* sporozoite surface-exposed invasion and migratory proteins used in alignments.

	TRSP	PBLP	SSP3	SIAP1	MAEBL	RON4	Falstatin	P113	PLP1/SPECT2	RON3
Pb	A1X5V6-1	A0A113RBV9								
Pb ANKA	A0A077X8K3	PBANKA_071220	PBANKA_1425200	PBANKA_1006200	PBANKA_0901300.2	PBANKA_0932000	PBANKA_0813000	PBANKA_1022500	PBANKA_1006300	PBANKA_1464900
Pch	A0A077TLB8		PCHAS_1427000	PCHAS_1007100	PCHAS_0702500	PCHAS_0912300	PCHAS_0813300	PCHAS_1023300	PCHAS_1007200	PCHAS_1467100
Pcy				PCYB_031630	PCYB_095300	PCYB_092210		PCYB_133650		
Pf 3D7	Q8I2A0	PF3D7_0818600	PF3D7_0812300	PF3D7_0408600	PF3D7_1147800.2	PF3D7_1116000	PF3D7_0911900	PF3D7_1420700	PF3D7_0408700	PF3D7_1252100
Pf IT		PFIT_0821500	PFIT_0815200	PFIT_0407200	PFIT_1148400.1	PFIT_1116800	PFIT_0912100	PFIT_1421700	PFIT_0407300	PFIT_1252300
Pg	A0A151LWY9	PGSY75_0818600								
Pk		PKH_051320	PKNH_1428300	PKNH_0306600	PKNH_0945700	PKNH_0913700	PKNH_0709900	PKNH_1337300	PKNH_0306700	PKNH_1472100
Pr	A0A060RRJ1		PRCDC_0811700	PRCDC_0406000	PRCDC_1146100.1		PRCDC_0910000	PRCDC_1420000	PRCDC_0406100	PRCDC_1251500
Pv		PVX_089485	PVX_123155	PVX_000815	PVX_092975	PVX_091434	PVX_099035	PVX_085445	PVX_000810	PVX_101485
Py					AF031886.2					
Py 17X			PY17X_1427200	PY17X_1007600	PY17X_0902700.1	PY17X_0934000	PY17X_0816300	PY17X_1024400	PY17X_1007700	PY17X_1467600
Py 17XNL				PY00455	PY05977					
Py YM			PYYM_1429000	PYYM_1007600	PYYM_0902200.1	PYYM_0933400	PYYM_0816000	PYYM_1024100	PYYM_1007700	PYYM_1469000

*Py* Plasmodium yoelii, *Pf IT* P. falciparum IT, *Pr* Plasmodium reichenowi, *Pv* P. vivax, *Pcy* P. cynomolgi, *Pk* P. knowlesi, *Pb* P. berghei, *Py 17XNL, 17X, YM* P. yoelli 17XNL, 17X, YM, *Pch* P. chabaudi, *Pf 3D7* P. falciparum 3D7, *Pg* P. gaboni, *Pb ANKA* P. berghei ANKA.

### Statistical tests used

GraphPad Prism (MacOS v6) and Microsoft Excel were used for all statistical analyses performed. Student’s *t*-test and ANOVA with Bonferroni’s multiple comparisons test were used on parametric data comparing two or more groups respectively. Log-rank (Mantel-Cox) tests were used to determine significant differences between survival curves.

### Reporting summary

Further information on experimental design is available in the Nature Research Reporting Summary linked to this paper.

## Supplementary information

Reporting Summary Checklist

## Data Availability

The data sets generated during and/or analysed during the current study are available from the corresponding author on reasonable request.
